# Effect of swap disorder on the physical properties of the quaternary Heusler alloy PdMnTiAl: a first-principles study

**DOI:** 10.1107/S205225251700745X

**Published:** 2017-06-21

**Authors:** Guanhua Qin, Wei Wu, Shunbo Hu, Yongxue Tao, Xiaoyan Yan, Chao Jing, Xi Li, Hui Gu, Shixun Cao, Wei Ren

**Affiliations:** a Physics Department and International Centre for Quantum and Molecular Structures, Shanghai University, Shanghai 200444, People’s Republic of China; b Materials Genome Institute and Shanghai Key Laboratory of High-Temperature Superconductors, Shanghai University, Shanghai 200444, People’s Republic of China; c State Key Laboratory of Advanced Special Steel, Shanghai University, Shanghai 200072, People’s Republic of China

**Keywords:** quaternary Heusler alloy, electronic properties, magnetic properties, swap disorder

## Abstract

Heusler alloys crystallize in a close-packed cubic structure, having a four-atom basis forming a face-centred cubic lattice. By selecting different composite elements, Heusler alloys provide a large family of members for frontier research of spintronics and magnetic materials and devices. In this paper, the structural, electronic and magnetic properties of a novel quaternary Heusler alloy, PdMnTiAl, have been investigated using a first-principles computational materials calculation. It was found that the stable ordered structure is a non-magnetic *Y*-type1, in good agreement with the Slater–Pauling rule. From the band structure and the density of states, it is predicted that this *Y*-type1 configuration is a new gapless semi-metal material. Furthermore, it was discovered that the Pd–Mn swap-disordered structure is more stable than the *Y*-type1 structure. The present work provides a guide for experiments to synthesize and characterize this Heusler alloy.

## Abstract 2

The effect of swap disorder on the physical properties of the quaternary Heusler alloy PdMnTiAl is described from first principles.

## 1. Introduction   

In recent years, spintronic materials and devices have been intensively investigated because of their great potential for information technology applications (Prinz, 1998 ▸). At the same time, the development of computational modelling techniques in materials science has triggered the study of a huge variety of magnetic materials such as Heusler compounds (Özdoğan *et al.*, 2013 ▸). Since 1903 (Heusler, 1903 ▸), when the first Heusler compound was synthesized, their intriguing physical properties, *e.g.* spintronic, optoelectronic and thermoelectric effects, have attracted the attention of numerous researchers (Zhang *et al.*, 2004 ▸; Nikolaev *et al.*, 2009 ▸; Abid *et al.*, 2016 ▸; Zhang *et al.*, 2015 ▸). Many novel materials have been synthesized experimentally and investigated theoretically (Jamer *et al.*, 2015 ▸; Fang *et al.*, 2014 ▸; Gao *et al.*, 2015 ▸; Ouardi *et al.*, 2013 ▸).

In regular Heusler *X*_2_ *YZ* compounds, *X* and *Y* are transition metals or rare earth elements and *Z* belongs to the main group of elements. There are generally two types of structure, Cu_2_MnAl with space group 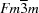 (225) and Hg_2_CuTi with space group 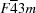 (216) (Graf *et al.*, 2011 ▸; Mohanta *et al.*, 2017 ▸). Half-Heusler alloys (*XYZ*) represent another large class of this family of materials and may crystallize in a non-centrosymmetric cubic space group, 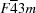 (Rogl *et al.*, 2016 ▸). The quaternary Heusler compounds *XX*′*YZ*, the structural prototype of which is the alloy LiMgPdSn, denoted as a *Y* structure, have recently been intensively investigated (Eberz *et al.*, 1980 ▸; Alijani *et al.*, 2011 ▸). The valence of *X*′ is lower than that of the *X* atom, and the valence of the *Y* element is lower than the valence of either *X* or *X*′. The sequence of atoms along the face-centred cube’s diagonal is *X*–*Y*–*X*′–*Z* and this was found to be the most energetically stable (Alijani *et al.*, 2011 ▸), called a *Y*-type1 phase, with fractional co­ordinates *X* 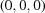, *X*′ 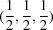, *Y* 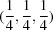 and *Z* 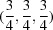. There are a further two types of atomic arrangement in the quaternary Heusler *XX*′*YZ* compound, namely *X*–*X*′–*Y*–*Z* *Y*-type2: *X* 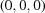, *X*′ 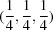, *Y* 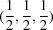 and *Z* 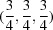, and *X*′–*Y*–*X*–*Z* *Y*-type3: *X* 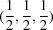, *X*′ 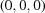, *Y* 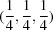 and *Z* 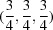 (Benkaddour *et al.*, 2016 ▸).

Previous studies of quaternary Heusler alloys (Galanakis *et al.*, 2016 ▸) showed interesting phenomena such as high spin polarization, half-metallic spin-gapless semiconductors and zero-gap material behaviours, which provide potential applications in spintronics, electronics and sensors (Wang *et al.*, 2010 ▸; Wang, 2008 ▸; Wang, Cheng, Wang, Wang & Liu, 2016 ▸; Wang, Cheng, Wang & Liu *et al.*, 2016 ▸). It has been found that the physical properties of Heusler alloys are highly dependent on the ordering of their structures (Graf *et al.*, 2011 ▸; Liu *et al.*, 2015 ▸). From experimental work, an unknown second phase precipitates in some Heusler alloys (Umetsu *et al.*, 2012 ▸). An interesting question is how the disordered structure influences the magnetic properties of a Heusler alloy. To answer this question, we systematically investigated disordered configurations and magnetic structures. Ti_2_Mn*Z* compounds were found to be half-metallic ferrimagnets with potential application in spintronic devices (Skaftouros *et al.*, 2013 ▸; Lukashev *et al.*, 2016 ▸; Fang *et al.*, 2014 ▸). In the present work, a new quaternary Heusler alloy, PdMnTiAl, has been designed on the basis of first-principles calculations. We studied both ordered and disordered structures of this material, and found this compound to be most stable when ordered in a *Y*-type1 structure. By using *VASP* and *AkaiKKR* calculations (see section 2 for more details), we found the ordered PdMnTiAl alloy to be a nonmagnetic material in a *Y*-type1 ground state, in good agreement with the Slater–Pauling rule. Interestingly, we revealed that the Pd–Mn swap-disordered structure (Picozzi *et al.*, 2004 ▸; Zhang *et al.*, 2013 ▸) is more stable than the *Y*-type1 structure, and may present different magnetic moments that can be tuned by the degree of swap disorder.

## 2. Computational methods   

The first-principles calculations were performed using the *Vienna *Ab initio* Simulation Package* (*VASP*) (Kresse & Furthmüller, 1996 ▸) within density functional theory (DFT). The ideal ordered quaternary Heusler alloy PdMnTiAl was built into a 16-atom supercell (four formula units). The geometry was then optimized to attain minimal energy structures. The energy cut-off was set to 500 eV. The convergences of energy and force were set to 10^−6^ eV and 0.01 eV Å^−1^, respectively. In our work, the exchange-correlation interaction is described by the Perdew–Burke–Ernzerhof (PBE) generalized gradient approximation (GGA) (Perdew *et al.*, 1996 ▸). The Brillouin zone was sampled using a set of 7 × 7 × 7 *k*-point mesh. We tested the *VASP* calculations by including the spin orbital coupling (SOC), and the results show that it has negligible influence on subsequent results in the present work, including the calculated total energy and magnetic moment of the studied compounds.

To explore the influence of atomistic disordering on the properties of the alloy, we used the program package *AkaiKKR* (Akai, 1992 ▸; Durham *et al.*, 1980 ▸), which utilizes the Korringa–Kohn–Rostoker (KKR) Green’s function method and has a high speed and high precision for systems with random distributions of atoms at given sites. For disordered systems, the coherent potential approximation (CPA) is one of the most efficient solution methods for averaged properties. Here, the configuration average of the Green’s function is expressed in terms of the effective mean atomic weight. Both ordered and disordered structures of the alloy were simulated by *AkaiKKR* calculations, and the ordered results were compared with the *VASP* predictions. The exchange-correlation effects were included by using a GGA91 scheme in the *AkaiKKR* calculations.

## 3. Results and discussion   

**Figure 1:**
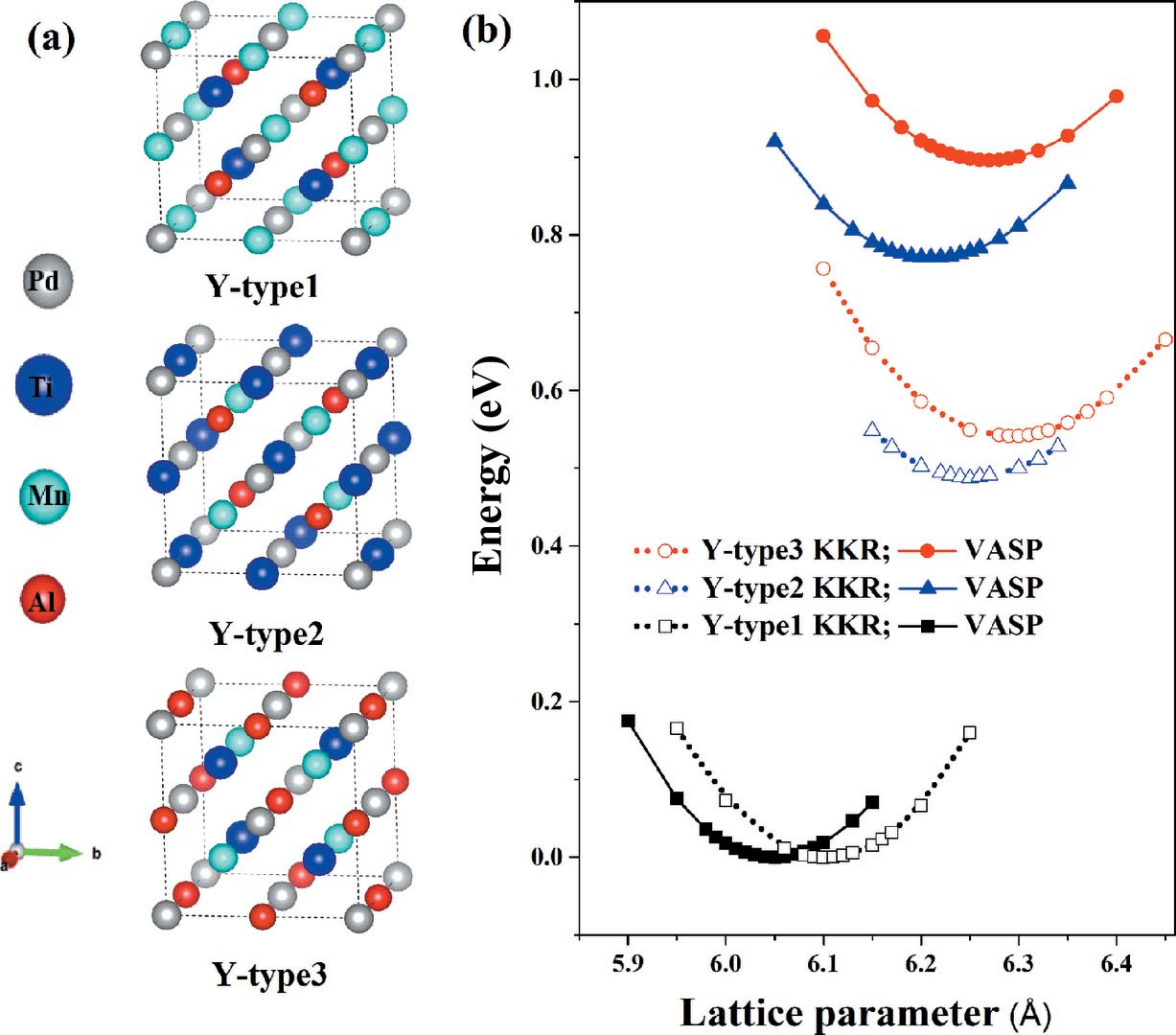
(*a*) The *Y*-type1, *Y*-type2 and *Y*-type3 crystal structures of the ordered PdMnTiAl Heusler alloys. The grey, blue, cyan and red spheres represent the elements Pd, Ti, Mn and Al occupying the positions *A* (0, 0, 0), *B* (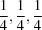), *C* (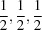) and *D* (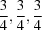), respectively. (*b*) The total energies per formula unit for different lattice parameters are obtained from geometry optimization using *AkaiKKR* (open symbols) and *VASP* (filled symbols).

First, the ordered quaternary Heusler alloy PdMnTiAl is considered. Fig. 1 ▸(*a*) shows the three PdMnTiAl structures with their different atomic arrangements. The *Y*-type1 structure with space group 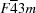 (No. 216) has an optimized lattice parameter of 6.05 Å. From Fig. 1 ▸(*b*), the *Y*-type2 and *Y*-type3 configurations are found from our calculations to have relatively higher total energies. Both *VASP* and *AkaiKKR* were used to optimize the structures of these three types of alloy to obtain the equilibrium lattice parameters. Similar trends were observed, such that the *Y*-type1 configuration had the smallest lattice parameter and *Y*-type3 the largest. A comparison of the two different calculation methods is shown in Table 1 ▸, together with the *VASP* and *AkaiKKR* predictions for the magnetic moments. The *Y*-type1 configuration of PdMnTiAl was found to have zero magnetization by both methods. To confirm the DFT calculation results, we checked the magnetic moments with the Slater–Pauling (SP) rule (Galanakis *et al.*, 2002 ▸) given by 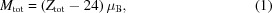
where *M*_tot_ is the total magnetic moment and *Z*_tot_ is the total number of valence electrons in the compound. The elements studied here have the following valence electron configurations: Pd (*s* ^2^ *d* ^8^), Ti (*s* ^2^ *d* ^2^), Mn (*s* ^2^ *d* ^5^) and Al (*s* ^2^ *p* ^1^). Thus the structure of our *Y*-type1 configuration complies perfectly with the Slater–Pauling rule.

**Figure 2:**
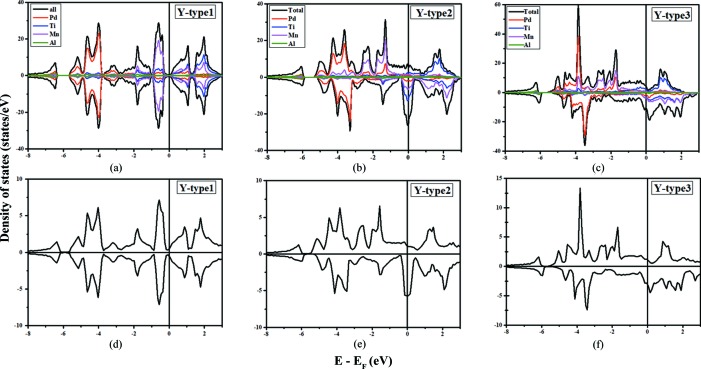
A comparison of the density of states (DOS) of three ordered PdMnTiAl structures from calculations using (*a*)–(*c*) *VASP* and (*d*)–(*f*) *AkaiKKR*. The projected DOS results are shown in panels (*a*)–(*c*) and the same results are obtained from both the *VASP* and *AkaiKKR* packages.

**Figure 3:**
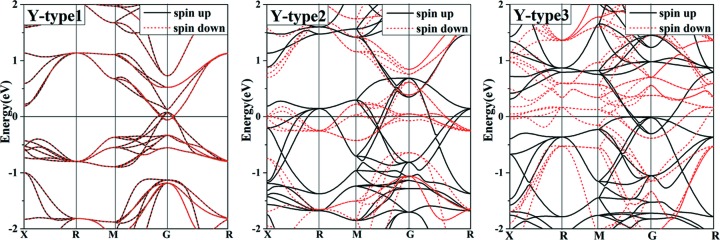
The calculated band structures of three ordered PdMnTiAl configurations based on *VASP* calculations. The Fermi level is set at zero energy.

From the optimized structures, we calculated their density of states (DOS) and band structures using *VASP* and *AkaiKKR*. Figs. 2 ▸ and 3 ▸ show that we obtain the same resulting electronic structures using *AkaiKKR* as with *VASP*. Fig. 2 ▸ shows that all three *Y*-type structures have bands which cross the Fermi level, thus indicating metallic behaviour. The nonmagnetic *Y*-type1 structure has a pseudo-gap-like DOS at the Fermi level, indicating a semi-metal, whereas the magnetic structures *Y*-type2 and *Y*-type3 have Fermi levels located near DOS peaks. This may help to explain the energetic instability of the *Y*-type2 and *Y*-type3 configurations. In Fig. 3 ▸, we present the band structures which correspond to the DOS results.

**Figure 4:**
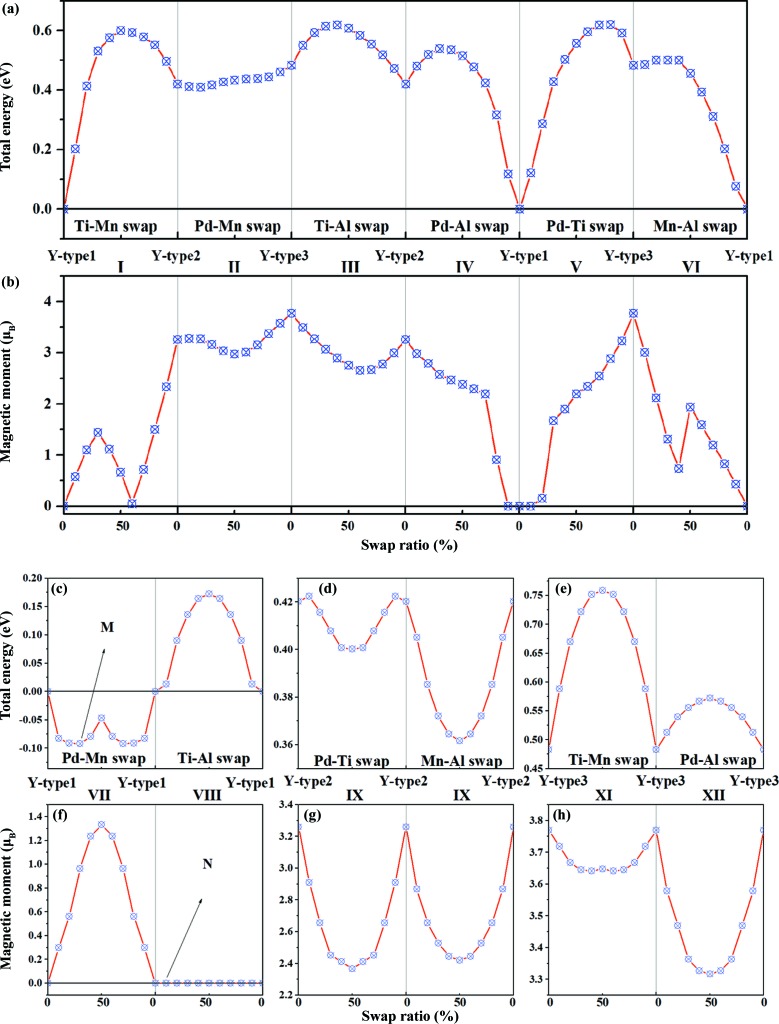
The total energy and magnetic moment of the disordered structures of PdMnTiAl Heusler alloys from *AkaiKKR* calculation. The swap disorder degrees of 10% to 90% indicate the element swap ratios, *e.g.* at point *N*, 10% means Pd(Ti_0.9_Al_0.1_)Mn(Al_0.9_Ti_0.1_). Panels (*a*) and (*c*)–(*e*) show the total energy values of different disordered structures, while panels (*b*) and (*f*)–(*h*) show the magnetic moments of different disordered structures. The top two panels (*a*) and (*b*) are for structures I–VI, while the bottom smaller panels (*c*)–(*h*) are for structures VII–XII.

From the above calculations the ordered *Y*-type structures have different total energies and magnetic moments, and PdMnTiAl is more likely to have the *Y*-type1 structure. The *Y*-type2 and *Y*-type3 structures have higher energy, larger lattice parameters and greater magnetic moments than the *Y*-type1 structure. To investigate the atomistic disordered configurations of these quaternary Heusler alloys further, we carried out advanced calculations using *AkaiKKR*. Based on the above ordered structures, we considered a number of possible swap disorder types by intermixing between any two of the Pd, Ti, Mn and Al atoms. In the following, we use the numerals I, II, III, IV, V and VI to represent interchanges, or swaps, between Ti–Mn, Pd–Mn, Ti–Al, Pd–Al, Pd–Ti and Mn–Al, respectively, and VII, VIII, IX, X, XI and XII to represent Pd–Mn, Ti–Al, Pd–Ti, Mn–Al, Ti–Mn and Pd–Al swaps, respectively. For example, the *M* point in Fig. 4 ▸ indicates a disordered configuration of (Pd_0.7_Mn_0.3_)Ti(Mn_0.7_Pd_0.3_)Al with a Pd–Mn swap. Fig. 4 ▸ shows the calculated total energy and the magnetic moment of all these different disordered structures. Surprisingly, the Pd–Mn swap-disordered structure is energetically more stable than the ordered *Y*-type1. Moreover, the Pd–Mn swap not only lowers the total energy, but also gives rise to significant magnetization. The new ground-state disordered structure (Pd_0.7_Mn_0.3_)Ti(Mn_0.7_Pd_0.3_)Al has an energy decrease of 0.092 eV per formula unit compared with the ordered *Y*-type1 PdMnTiAl, with its total magnetic moment enhanced to 0.964 µ_B_. The half-and-half Pd–Mn randomly disordered configuration (Pd_0.5_Mn_0.5_)Ti(Mn_0.5_Pd_0.5_)Al is 0.047 eV lower in energy per formula unit and has the maximum magnetic moment of 1.132 µ_B_. In the other cases of disorder, the total energies are increased by the disordering effect and the swap disorders tend to introduce finite total magnetization. We also calculated the DOS for all the different dis­ordered structures, as presented in Fig. S1 in the supporting information.

We attempted to construct larger supercells and to use *VASP* to verify the validity of the corresponding *AkaiKKR* results. In a double-sized supercell with 32 atoms (eight formula units), 25% swap disorders were simulated by exchanging two of the eight Pd atom positions and two of the eight Mn atom positions. Similarly, 50% swap disorders were simulated by exchanging four of the eight Pd atoms and four of the eight Mn atom positions. Four possible disordered supercell structures for the 50% configurational swap disorder are shown in Fig. S2 in the supporting information. We found the swap-disordered structures to be more stable than the ordered PdMnTiAl *Y*-type1 structure and have also verified qualitatively the correctness of the magnetic moments. The results, shown in Table S1 in the supporting information, suggest that much larger supercells might be necessary to achieve a better quantitative comparison between *AkaiKKR* disorder calculations and *VASP* supercell calculations. This comparison is beyond our current computational resources. However, experimental work is expected to synthesize and characterize the proposed PdMnTiAl quaternary Heusler alloy for eventual confirmation of our prediction.

## 4. Conclusions   

The structure and electronic and magnetic properties of the quaternary Heusler alloy PdMnTiAl have been investigated by first-principles calculations. In these compounds, the ordered configuration of *Y*-type1 is more stable than those of *Y*-type2 and *Y*-type3. The semi-metallic *Y*-type1 configuration shows zero magnetic moment, in good agreement with the Slater–Pauling rule. Interestingly, we discovered that the Pd–Mn swap-disordered structure is more stable than the ordered *Y*-type1 configuration, and that the total magnetizations of these disordered (Pd_1−*x*_Mn_*x*_)Ti(Mn_1−*x*_Pd_*x*_)Al compounds are dependent on the degree of Pd–Mn swap, 0 < *x* < 1. We hope these findings will stimulate further investigation into spintronics materials and devices.

## Supplementary Material

Additional table and figures. DOI: 10.1107/S205225251700745X/ct5004sup1.pdf

## Figures and Tables

**Table 1:** A comparison of two computational methods for ordered structures (*VASP* and *AkaiKKR*) The *Y*-type1 structure with the lowest energy and magnetic moment (PdMnTiAl) is used as a reference.

		*a* (Å)	Δ*E* (eV)	Magnetic moment (μ_B_)	*m*_[*X*]_	*m*_[*X*′]_	*m*_[*Y*]_	*m*_[*Z*]_
*VASP*	*Y*-type1	6.05	0	0	0	0	0	0
	*Y*-type2	6.14	0.772	2.59	0.192	3.055	−0.676	−0.019
	*Y*-type3	6.21	0.897	3.87	0.160	3.567	0.182	−0.036
*AkaiKKR*	*Y*-type1	6.10	0	0	0	0	0	0
	*Y*-type2	6.25	0.420	3.26	0.162	3.554	−0.433	−0.043
	*Y*-type3	6.30	0.483	3.84	0.130	3.650	0.040	−0.059
