# Supplementary material for: Effect of swap disorder on the physical properties of the quaternary Heusler alloy PdMnTiAl: a first-principles study
Source: IUCrJ. 2017 Jun 21;4(Pt 4):506–11. doi: 10.1107/S205225251700745X (PMC5571813; doi:10.1107/S205225251700745X)
Supplement: Supplementary file 1 [file m-04-00506-sup1.pdf]

IUCrJ

Volume 4 (2017)

Supporting information for article:

Effect of swap disorder on the physical properties of the quaternary Heusler alloy PdMnTiAl: a first-principles study

Guanhua Qin, Wei Wu, Shunbo Hu, Yongxue Tao, Xiaoyan Yan, Chao Jing, Xi Li, Hui Gu, Shixun Cao and Wei Ren

Supplementary Materials

Swap disorder effect on the physical properties of quaternary Heusler alloy PdMnTiAl: a first-principles study

Guanhua Qin[1,2], Wei Wu[1,2], Shunbo Hu[1,2], Yongxue Tao[1], Xiaoyan Yan[1], Chao Jing[1], Xi Li[3], Hui Gu[2], Shixun Cao[1,2] and Wei Ren[1,2,*]

[1] Physics Department, and International Centre for Quantum and Molecular Structures, Shanghai University, Shanghai 200444, China

[2] Materials Genome Institute, and Shanghai Key Laboratory of High Temperature Superconductors, Shanghai University, Shanghai 200444, China

[3] State Key Laboratory of Advanced Special Steel, Shanghai University, Shanghai 200072, China

*renwei@shu.edu.cn

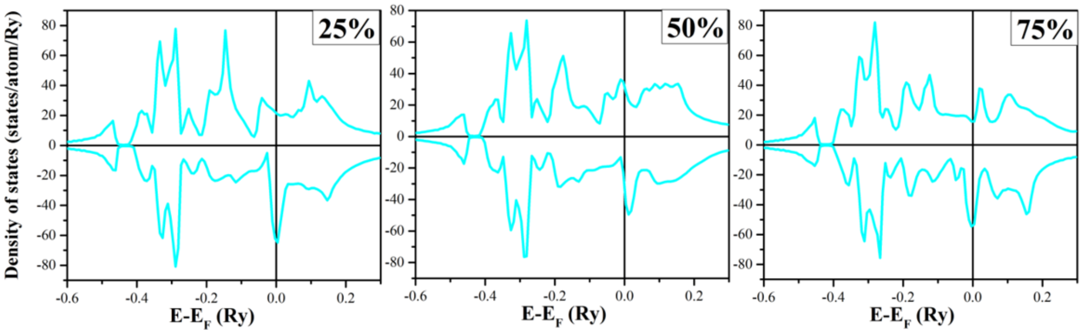

(a) $Pd(Ti_{1-x}Mn_x)(Mn_{1-x}Ti_x)Al$

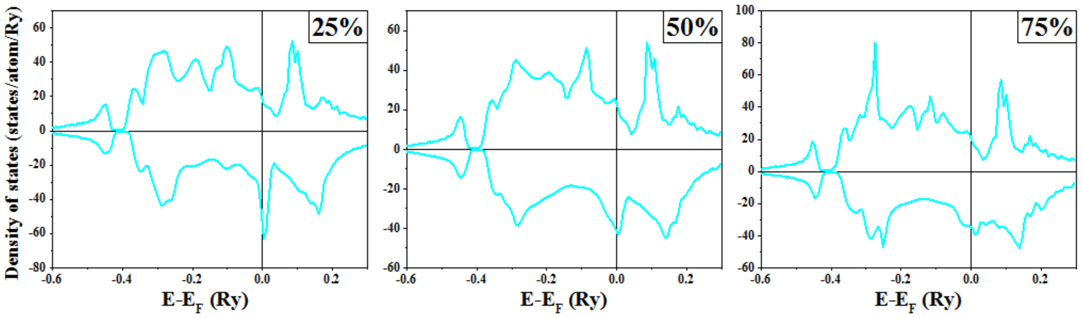

(b) $(Pd_{1-x}Mn_x)(Mn_{1-x}Pd_x)TiAl$

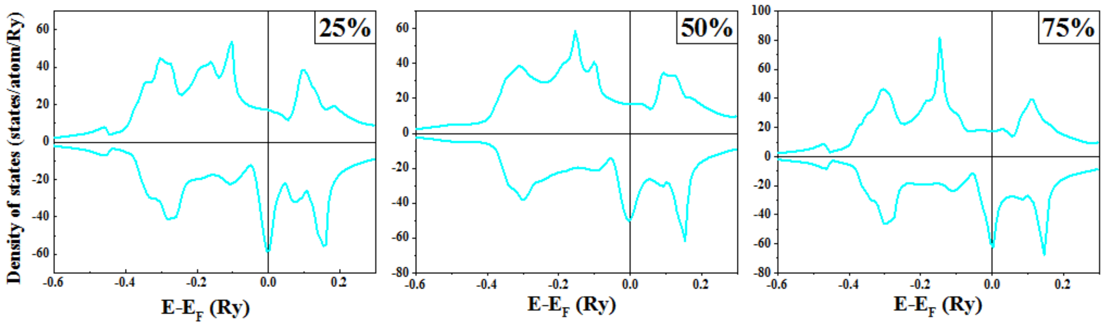

(c) $PdMn(Al_{1-x}Ti_x)(Ti_{1-x}Al_x)$

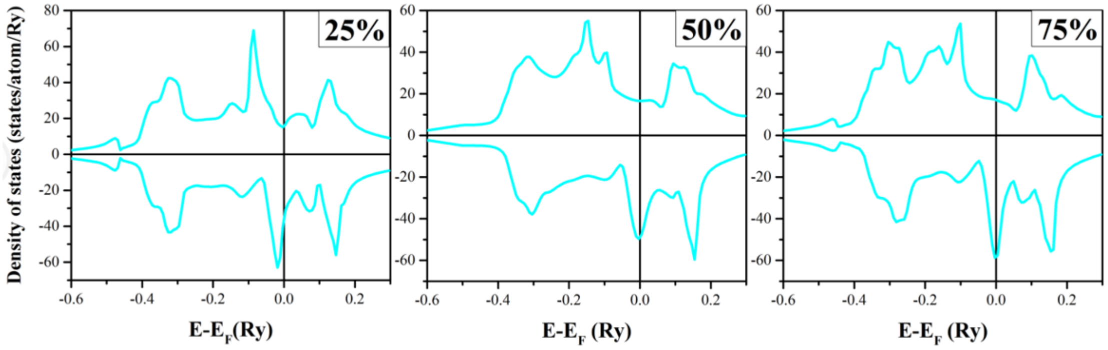

(d) $(Pd_{1-x}Al_x)MnTi(Al_{1-x}Pd_x)$

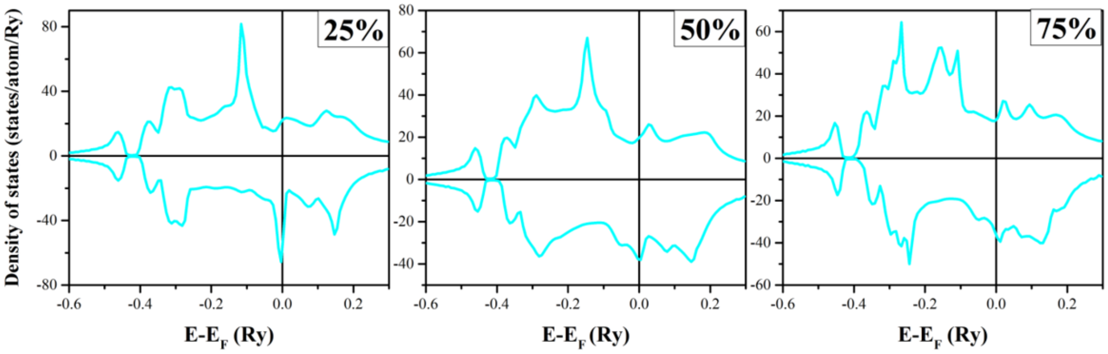

(e) $(Pd_{1-x}Ti_x)(Ti_{1-x}Pd_x)MnAl$

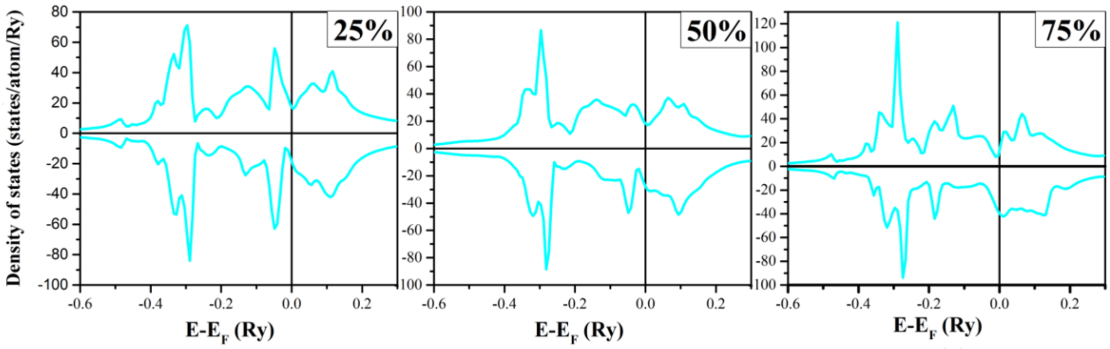

(f) $Pd(Mn_{1-x}Al_x)(Al_{1-x}Mn_x)Ti$

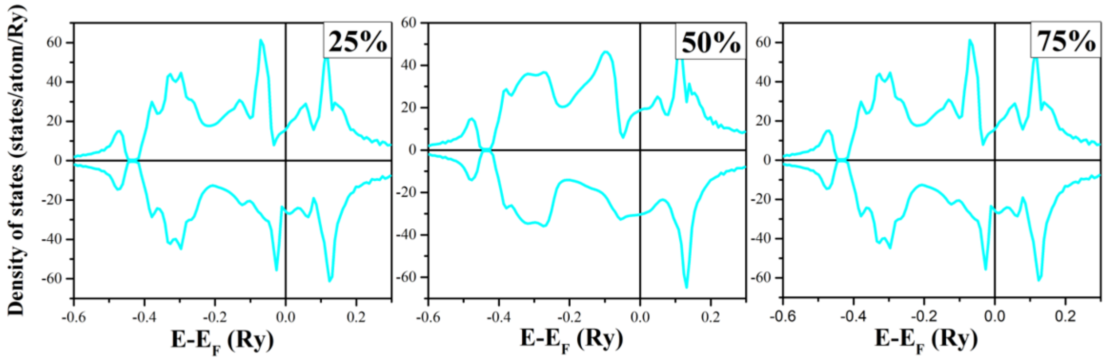

(g) $(Pd_{1-x}Mn_x)Ti(Mn_{1-x}Pd_x)Al$

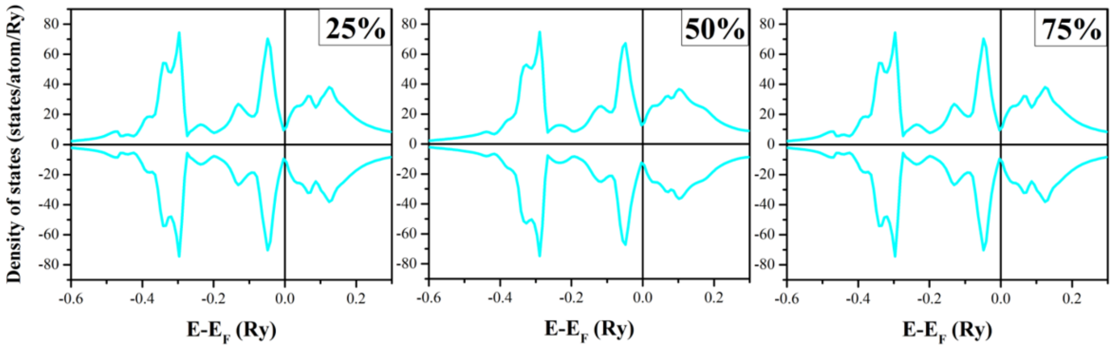

(h) $Pd(Ti_{1-x}Al_x)Mn(Al_{1-x}Ti_x)$

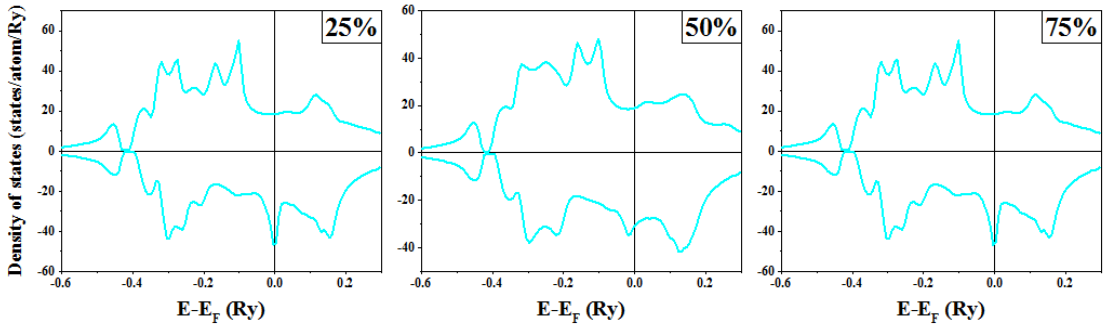

(i) $(Pd_{1-x}Ti_x)Mn(Ti_{1-x}Pd_x)Al$

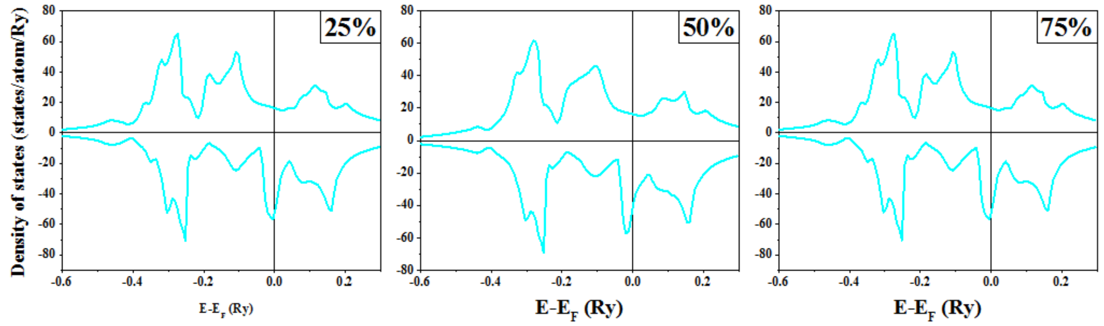

(j) $Pd(Mn_{1-x}Al_x)Ti(Al_{1-x}Mn_x)$

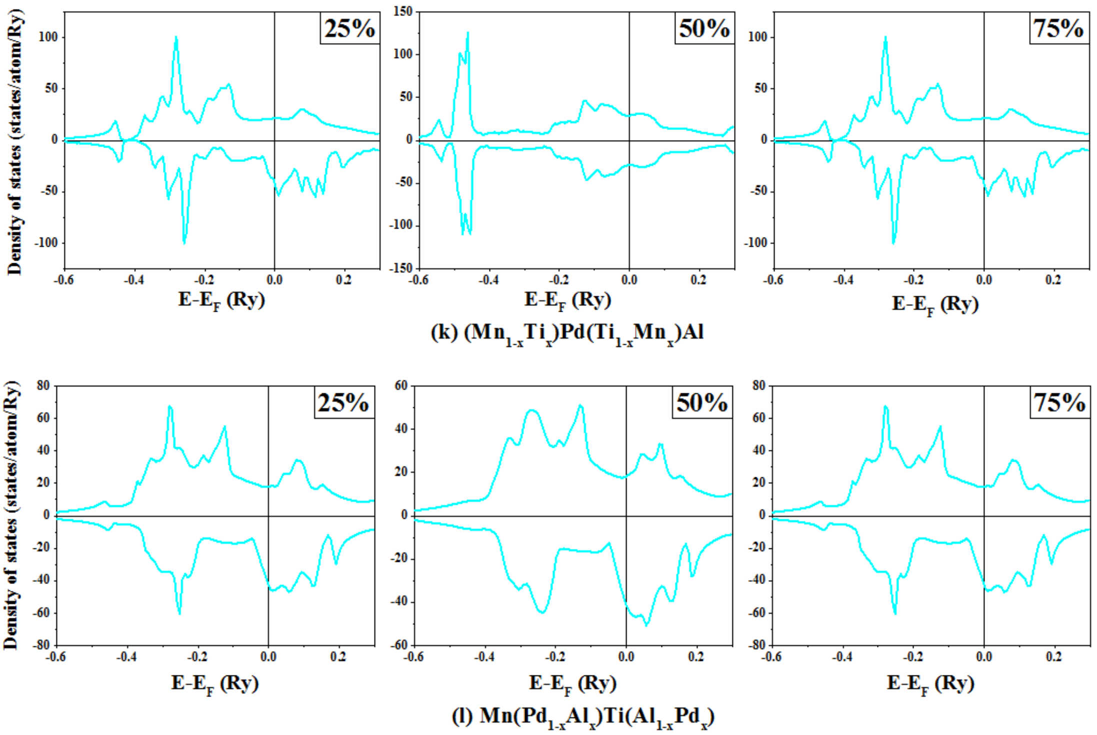

Figure S1 The density of states (DOS) of disordered structures. We present the disorder degrees of x=25%, 50% and 75% with (a) Ti-Mn sawp, (b) Pd-Mn swap, (c) Ti-Al swap, (d) Mn-Al swap, (e) Pd-Mn swap, (f) Ti-Al swap, (g) Pd-Mn swap, (h)Ti-Al swap, (i) Pd-Ti swap, (j) Mn-Al swap, (k) Ti-Mn swap, and (l) Pd-Al swap.

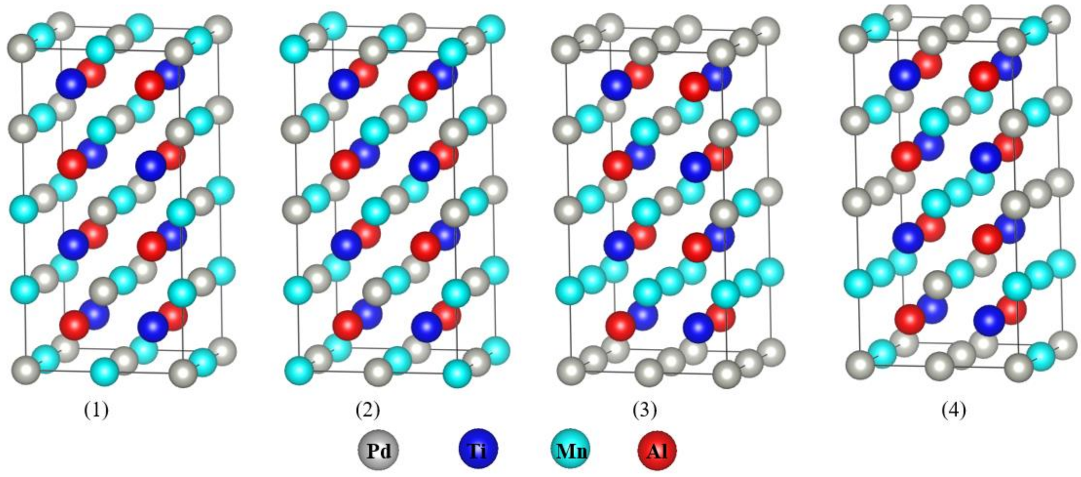

Figure S2 The different structures of 50% swap disorder in VASP supercell simulation of “disordered” structure. The grey, blue, cyan and red spheres are for Pd, Ti, Mn, and Al elements respectively.

Table S1 The results of Pd-Mn swap disorder simulations using VASP and AkaiKKR. (The lowest energy is set as zero.)

	Swap ratio	Total energy	Magnetic moment
VASP	0%	0eV	$0\mu_B$
	25%	-0.377eV	$0.440\mu_B$
	50%(1)	-0.080eV	$0.120\mu_B$
	50%(2)	-0.043eV	$0.108\mu_B$
	50%(3)	-0.248eV	$0.175\mu_B$
	50%(4)	-0.228eV	$0.574\mu_B$
AkaiKKR	0%	0eV	$0\mu_B$
	25%	-0.092eV	$0.803\mu_B$
	50%	-0.047eV	$1.132\mu_B$
